# Supplementary material for: Loss of Mature Lamin A/C Triggers a Shift in Intracellular Metabolic Homeostasis via AMPKα Activation
Source: Cells. 2022 Dec 9;11(24):3988. doi: 10.3390/cells11243988 (PMC9777081; doi:10.3390/cells11243988)
Supplement: Supplementary file 1 [file cells-11-03988-s001.zip › revised Suppl fig.rtf]

Supplemental Figure


Figure S1 Western blot detected the expression of lamin A/C, pLamin A/C and lipid metabolism synthesis-related proteins in obese rat models. All western blot pictures (n=5) in fat tissues (A) and liver tissues of obese rat (B).¡£


Figure S2. LMNA-deletion in 7701 cells downregulated the expression of MPC1 and MPC2 and thus induced lactate accumulation. (A) The expression levels of MPC1 and MPC2 in LMNA-KO 7701 cells are shown with the corresponding quantitative plot (n=5) (B). (C) qRT-PCR was utilized to examine the mRNA expression of ACC1 and MPC1 and MPC2 in LMNA-KO 7701 cells (n=3). Data are shown as the mean ± SD. ns, p > 0.05, * p < 0.05, ** p < 0.01.


Figure S3. Lamin C interacted with pAMPKá-172. (A) Co-IP assays were performed to assess the endogenous interaction between pLamin A/C and pACC1 and pLamin A/C and FASN in 7701 cells. IgG was used as a negative control, and the input was used to examine the levels of FASN, pACC1, and pLamin A. (E) HEK293T cells were transiently co-transfected with the plasmid containing the LMNA cDNA and the FLAG- AMPKá-full length (AMPKá-F), -N (AMPKá-N) or -C termini ((AMPKá-C) plasmid or control vector. An anti-FLAG antibody was used to immunoprecipitate the cell lysates.


           


Figure S4.  LMNA and pLMNA from 3 cell lines were downregulated by AICAR in a concentration-dependent manner. 
