# Supplementary material for: Loss of Mature Lamin A/C Triggers a Shift in Intracellular Metabolic Homeostasis via AMPKα Activation
Source: Cells. 2022 Dec 9;11(24):3988. doi: 10.3390/cells11243988 (PMC9777081; doi:10.3390/cells11243988)
Supplement: Supplementary file 1 [file cells-11-03988-s001.zip › revised Table S1.docx]

**Table S1 Primers in experiments**

| Name | Human-*LMNA*-sgRNA primer (6 pairs)  Sequences |  |
| --- | --- | --- |
| sg*LMNA*-1F: | **5’- caccgGCGCCGTCATGAGACCCGAC - 3’** |  |
| sg*LMNA*-1R: | **5’- aaacGTCGGGTCTCATGACGGCGCc - 3’** |  |
|  |  |  |
| sg*LMNA*-2F: | **5’- caccgGCCGAGCCTGAGCAGCTATC - 3’** |  |
| sg*LMNA*-2R: | **5’- aaacGATAGCTGCTCAGGCTCGGCc - 3’** |  |
|  |  |  |
| sg*LMNA*-3F: | **5’- caccgCCTGCAGCGCATCCGCCAGC - 3’** |  |
| sg*LMNA*-3R: | **5’- aaacGCTGGCGGATGCGCTGCAGGc - 3’** |  |
|  |  |  |
| sg*LMNA*-4F: | **5’- caccgTCTCAGTGAGAAGCGCACGC - 3’** |  |
| sg*LMNA*-4R: | **5’- aaacGCGTGCGCTTCTCACTGAGAc - 3’** |  |
|  |  |  |
| sg*LMNA*-5F: | **5’- caccgCCAGAAGAACATCTACAGTG** **- 3’** |  |
| sg*LMNA*-5R: | **5’- aaacCACTGTAGATGTTCTTCTGGc - 3’** |  |
|  |  |  |
| sg*LMNA*-6F: | **5’- caccgCCTCACTGTAGATGTTCTTC - 3’** |  |
| sg*LMNA*-6R: | **5’- aaacGAAGAACATCTACAGTGAGGc -3’** |  |
|  |  |  |
|  | **Mouse-*LMNA*-shRNA primer (3 pairs)** |  |
| Name | **Sequences** | |
| shRNA-*LMNA*-1F: | **5’-ccggCAGTATAAGAAGGAGCTAGAActcgagTTCTAGCTCCTTCTTATACTGttttt -3’** | |
| shRNA-LMNA-1R: | **5’-aattaaaaaCAGTATAAGAAGGAGCTAGAActcgagTTCTAGCTCCTTCTTATACTG-3’** | |
|  |  | |
| shRNA-*LMNA*-2F: | **5’-ccggGACGATCCTTTGATGACCTATctcgagATAGGTCATCAAAGGATCGTCttttt-3’** | |
| shRNA-*LMNA*-2R: | **5’-aattaaaaaGACGATCCTTTGATGACCTATctcgagATAGGTCATCAAAGGATCGTC-3’** | |
|  |  | |
| shRNA-*LMNA*-3F: | **5’-ccggGCTTGACTTCCAGAAGAACATctcgagATGTTCTTCTGGAAGTCAAGCttttt -3’** | |
| shRNA-*LMNA*-3R: | **5’-aattaaaaaGCTTGACTTCCAGAAGAACATctcgagATGTTCTTCTGGAAGTCAAGC-3’** | |
|  |  | |
|  | **QRT-PCR primers** | |
| Name | **Sequences** | |
| ACC1-F: | **5’ -TCAATCTTGAGGGCTAGGTCTTT-3’** | |
| ACC1-R: | **5’ -GGTTCAGCTCCAGAGGTTGG-3’** | |
|  |  | |
| MPC1-F: | **5’- ACTATGTCCGAAGCAAGGATTTC****-3’** | |
| MPC1-R: | **5’- CGCCCACTGATAATCTCTGGAG****-3’** | |
|  |  | |
| MPC2-F: | **5’- TACCACCGGCTCCTCGATAAA****-3’** | |
| MPC2-R: | **5’- TATCAGCCAATCCAGCACACA-3’** | |
|  |  | |
| GAPDH-F： | **5’ -TCTCCTCTGACTTCAACAGCGAC****-3’** | |
| GAPDH-R： | **5’ - CCCTGTTGCTGTAGCCAAATTC** **-3’** | |
|  |  | |
|  | ***PreLMNA* and Mutation primers** | |

| Name | Sequences |
| --- | --- |
| PreLMNA | **F: CGTCGACTGGATCCGGTACCGAGGAGAT**  **R: CTCGAGCGGCCGCGTACGCGTCATGATGCTGCAGTTCT** |
| Mutation 1 (M1, D230N) | **F: ACTGGTGGAGATTAACAAT**  **R: GCTTCCCATTGTTAATCTCC** |
| mutation 2 (M2, G465D) | **F: GACCAGTCCATGGACAATT**  **R: ATCTGCCAATTGTCCATGGA** |
| Mutation 3 (M3, R482W) | **F: TTGCTGACTTACTTGTTCCCACC**  **R: GAACTTTGGTGGGAACAAGTAA** |
| mutation 4 (M4, T528R) | **F: TGCGTAGGGCTCTCATCAAC**  **R: TGAGAGCCCTACGCAGGC** |
